# Addressing Gender-Based Disparities in Earning Potential in Academic Medicine

**DOI:** 10.1001/jamanetworkopen.2022.0067

**Published:** 2022-02-18

**Authors:** Eva Catenaccio, Jonathan M. Rochlin, Harold K. Simon

**Affiliations:** 1 Division of Pediatric Neurology, Department of Pediatrics, The Children’s Hospital of Philadelphia, Philadelphia, Pennsylvania; 2 Division of Pediatric Emergency Medicine, Department of Emergency Medicine, Maimonides Medical Center, Brooklyn, New York; 3 Department of Pediatrics, Emory University School of Medicine, Children’s Healthcare of Atlanta, Atlanta, Georgia; 4 Department of Emergency Medicine, Emory University School of Medicine, Atlanta, Georgia

## Abstract

**Importance:**

Gender-based disparities in compensation in academic medicine are recognized, but their estimated impacts on early career earning potential and strategies to mitigate them have not been well studied.

**Objectives:**

To compare earning potential between female and male academic physicians in the first 10 years of posttraining employment and to evaluate the estimated impact of promotion timing, starting salary, and salary growth rate on earning potential.

**Design, Setting, and Participants:**

Using publicly available mean debt and compensation data for full-time employed academic physicians in the US from 2019 to 2020, starting salary, salary in year 10 of employment, annual salary growth rate, and overall earning potential in the first 10 years of employment were estimated for each gender by subspecialty. The estimated impacts of promotion timing and potential interventions, including equalizing starting salaries and annual salary growth rates, were modeled. Data analysis was performed from March to May 2021.

**Exposures:**

Gender and subspecialty.

**Main Outcomes and Measures:**

Starting salary, annual salary growth rate, year-10 salary, and earning potential in first 10 years of employment.

**Results:**

This cross-sectional study included compensation data from 24 593 female and 29 886 male academic physicians across 45 subspecialties. Women had lower starting salaries in 42 of 45 subspecialties (93%), year-10 salaries in 43 of 45 subspecialties (96%), mean annual salary growth rates in 22 of 45 subspecialties (49%), and earning potential in 43 of 45 subspecialties (96%) (median [IQR], $214 440 [$130 423-$384 954], or 10%, less). A 1-year delay in promotion from assistant to associate professor reduced women’s earning potential by a median (IQR) of $26 042 ($19 672-$35 671), but failure to be promoted at all reduced it by a median (IQR) of $218 724 ($176 317-$284 466). Equalizing starting salaries could increase women’s earning potential by a median (IQR) of $250 075 ($161 299-$381 799) in the subspecialties for which starting salaries for women were lower than those for men. Equalizing annual salary growth rates could increase women’s earning potential by a median (IQR) of $53 661 ($24 258-$102 892) in the subspecialties for which mean annual salary growth rates were lower for women than for men.

**Conclusions and Relevance:**

The findings of this study suggest that gender-based disparities in starting salary and early career earning potential are pervasive in academic medicine in the US. Equalizing starting salaries would address the majority of the differences in earning potential.

## Key Points

**Question:**

Are there gender-based differences in early career earning potential between female and male academic physicians in the US and, if so, are they predominantly the result of unequal starting salaries or annual salary growth rates?

**Findings:**

In this cross-sectional study of 54 479 academic physicians, women had lower starting salaries in 42 of 45 subspecialties, lower mean annual salary growth rates in 22 of 45 subspecialties, and lower 10-year earning potential in 43 of 45 subspecialties. Equalizing starting salaries would make up the majority of the gender-based differences in early career earning potential.

**Meaning:**

These findings suggest that gender-based disparities in early career earning potential are pervasive in academic medicine in the US but could be addressed by equalizing starting salaries.

## Teaser

This cross-sectional study compares earning potential between female and male academic physicians in the first 10 years of posttraining employment and evaluates the estimated impact of promotion timing, starting salary, and salary growth rate on earning potential.

## Introduction

In recent years, gender-based disparities in salary, academic promotion, and leadership attainment have been well documented across academic medicine in the US.^1,2,3,4,5,6,7,8,9^ Subspecialties that have a higher proportion of women, such as pediatrics, tend to be less well compensated than those that have a higher proportion of men, such as orthopedic surgery.^1,6^ Moreover, as the proportion of women in a subspecialty increases over time, compensation in that subspecialty has often decreased relative to other subspecialties, helping to support the theory of the devaluation of work that is seen as women’s work.^1,6^ Gender-based differences in salary are present at the start of medical careers^10,11^ and persist when accounting for age, experience, academic rank, and measures of research productivity and revenue generation.^4,12^ Although gender-based disparities in academic medicine have long been recognized as an issue, few studies have evaluated the cumulative impact of these disparities or have focused on modeling potential strategies to mitigate them.^13^

We previously demonstrated wide variation in earning potential between subspecialties in both pediatric and adult medicine and showed that these differences are associated with workforce distribution.^14,15,16,17^ Our objectives in the present study were to examine differences in early career earning potential between female and male academic physicians within subspecialties and to model potential strategies to mitigate them. We estimated annual salaries, annual salary growth rates, and overall earning potential in the first 10 years of posttraining employment for both female and male academic physicians across adult medical, adult surgical, and pediatric medical subspecialties. We directly compared women and men within subspecialties. We evaluated the role of promotion timing and the potential outcomes of equalizing starting salaries and mean annual salary growth rates across the genders as potential interventions to address gender-based disparities in early career earning potential.

## Methods

Because we used publicly available, aggregated, and deidentified data, this cross-sectional study did not meet the criteria for human participant research and did not require institutional review board approval or informed consent, in accordance with 45 CFR §46. This study follows the Strengthening the Reporting of Observational Studies in Epidemiology (STROBE) reporting guideline.

### Data Sources

We obtained information on subspecialty-specific mean compensation and educational debt for the academic year of July 2019 to June 2020. For postresidency and/or postfellowship subspecialty-specific compensation estimates by academic rank, we used data from the Association of American Medical Colleges (AAMC) annual Medical School Faculty Salary Survey report, which started reporting compensation data broken down by gender in 2019 to 2020.^18^ AAMC compensation data represent the mean fixed or contractual salary component of total compensation plus the supplemental earnings components of total compensation (medical practice supplement, bonus or incentive pay, and known uncontrolled outside earnings), before taxes and retirement and fringe benefits, of full-time faculty affiliated with Liaison Committee on Medical Education–accredited medical schools in the US. These data are reported as a single mean figure by rank and are reported in 2019 to 2020 US dollars. The 2019 to 2020 survey reflected data from 153 medical schools and had a response rate of more than 99%.^19^

We obtained mean educational debt from the AAMC 2020 Debt Fact Card.^20^ The differences between genders with respect to educational debt appear to be negligible^21^; however, we included educational debt as part of our models of earning potential to provide a more accurate estimate of the financial impact of different careers in medicine and to remain consistent with our prior publications.^14,15,16,17^ We assumed that loan repayment was deferred during training and then was repaid over 25 years and that the accrued interest was capitalized once training was completed. We used an interest rate of 6.08% on the basis of the rates of federal Stafford educational loans for 2019 to 2020.^22^

### Estimation of Annual Salaries and Early Career Earning Potential

As described in our prior reports,^15,16^ we used cross-sectional compensation-by-rank data to generate annual net income streams for female and male academic physicians over the first 10 years of employment after residency and/or fellowship. We assumed that, after completing training, graduates worked as assistant professors for 7 years before being promoted to associate professors.^23,24,25^ We assumed a within-rank salary growth rate equal to 50% of the between-rank salary growth rate calculated from AAMC data, with the within-rank mean salary being attained by the fourth year at each rank. On the basis of our projections, we estimated starting salary, defined as the salary in the first year of posttraining employment, and the salary at year 10 of employment (year-10 salary), meant to reflect an early to midcareer salary.

To compare early career earning potential between female and male physicians, we used the concept of the net present value (NPV), which is a standard financial technique used to analyze the value of different income streams over time.^14,26^ The NPV addresses the concept that income obtained today is more valuable than future income, because today’s income can be invested to yield an immediate return. The formula for NPV is as follows:
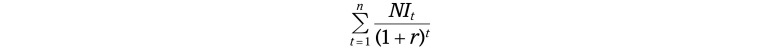
where *NI* is annual net income, which we defined as annual compensation less annual debt-repayment costs.^26^ The formula takes the sum of the annual net incomes over time (from *t* = 1 to *n* years) and discounts them back to the present at a discount rate (*r*), which was set at 3.0% according to the discount rate in July of 2019.^27^ From this calculation, one can compare the current value of future net income streams. We defined the 10-year NPV as the present value of the net income generated from a career in a subspecialty over the first 10 years of employment. The 10-year NPV is an estimate of the earning potential in the early part of a career, as it represents the financial returns that a graduating resident might expect in the first 10 years of employment from either entering general practice right after residency or pursuing fellowship training followed by practice as a subspecialist.

### Statistical Analysis

#### Sensitivity Analyses: Promotion Timing

We modeled the estimated financial impact of gender-based differences on promotion timing, because research has shown that women in academic medicine are promoted later and less frequently than men.^2,8,9^ A recent study^8^ found that women were less likely than men to be promoted to associate professor, and, for women who were promoted from assistant to associate professor, there was a median delay of 214 days compared with men. On the basis of these findings, we first modeled the scenario in which women were promoted from assistant to associate professors after 8 years, whereas men were promoted after 7 years. In the second scenario, women were not promoted within the initial 10-year period of their career and remained at the assistant professor rank, whereas men still were promoted from assistant to associate professor after 7 years.

#### Sensitivity Analyses: Interventions to Address Gender-Based Disparities in Early Career Earning Potential

We performed 2 additional sensitivity analyses to assess potential interventions to address gender-based disparities in early career earning potential and to evaluate the relative contribution of starting salary vs salary growth rate. First, we modeled increasing the starting salaries for women to match those of men in subspecialties in which women had lower estimated starting salaries while leaving gender-specific growth rates the same as in our original model. In the second sensitivity analysis, we set annual salary growth rates for women to match those of men in subspecialties in which the mean annual salary growth rate over the first 10 years of employment was lower for women but kept gender-specific starting salaries the same as in our original model.

Data were collected and analyzed with Excel software version 365 (Microsoft). Data analysis was performed from March to May 2021.

## Results

Our study included compensation data from 24 593 female and 29 886 male academic physicians across 21 adult medical, 8 adult surgical, and 16 pediatric medical subspecialties (eTable 1 in the Supplement). For the 2019 to 2020 AAMC Faculty Salary Survey, gender was reported for 95% of respondents and there was a mean of 303 individuals surveyed per academic rank per subspecialty for the assistant and associate professor levels.

**Figure 1.**
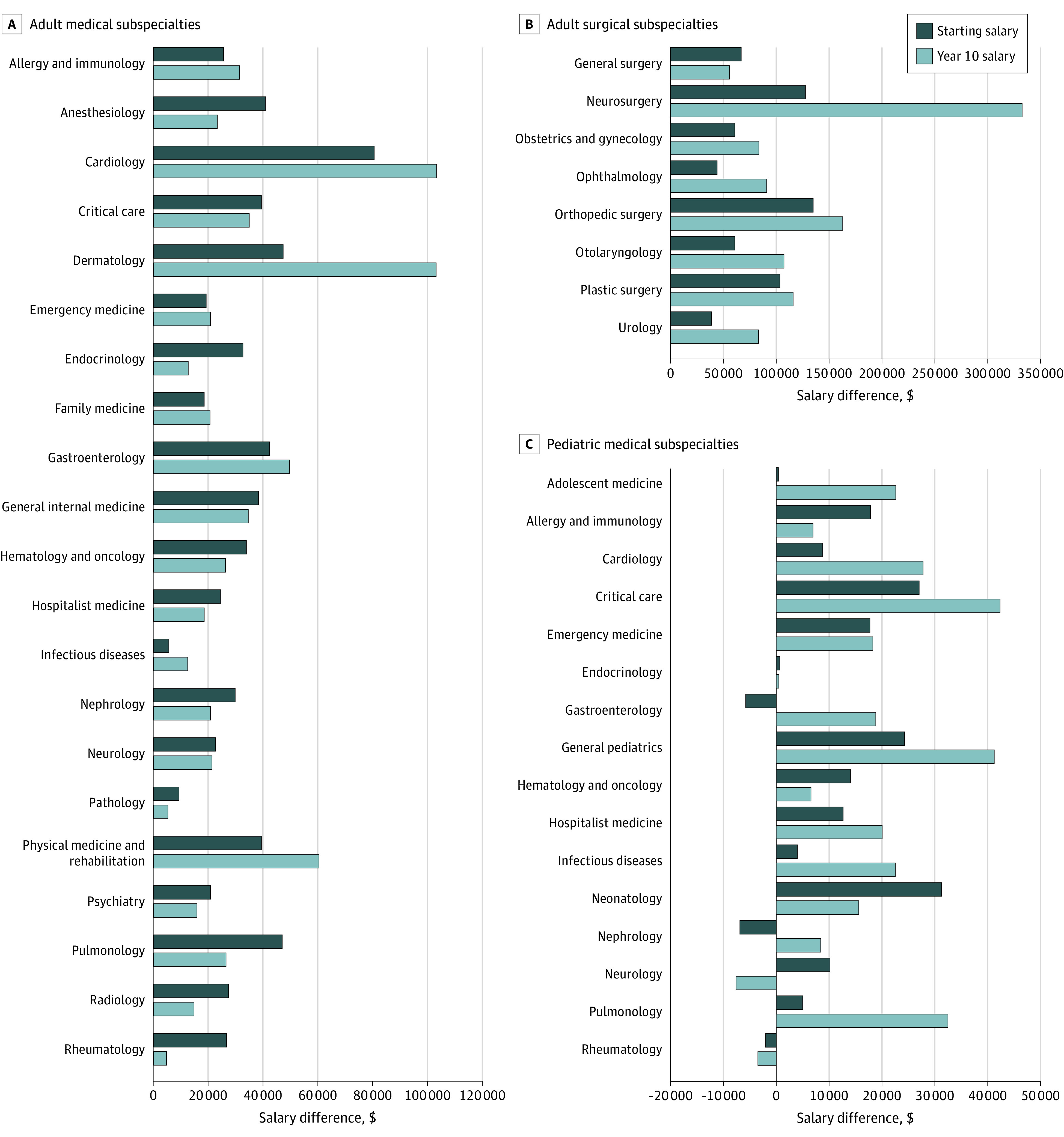
Gender Differences in Posttraining Starting Salary and Salary at Year 10 of Employment Graphs show data for adult medical (A), adult surgical (B), and pediatric medical (C) subspecialties. Positive values on the x-axis represent higher salaries for men. Negative values on the x-axis represent higher salaries for women.

For 42 of the 45 subspecialties (93%), women had a lower estimated starting salary than men (Table 1, Figure 1, and eTable 1 in the Supplement). For 43 of 45 subspecialties (96%), women had a lower estimated year-10 salary than men. Women had a starting salary that was a median (IQR) of $26 800 ($12 816-$40 980), or 10%, lower than that for men and a year-10 salary that was a median (IQR) of $22 890 ($15 808-$49 781), or 9%, lower than that for men. Differences in mean annual salary growth rates were more variable and more modest (Table 1 and eTable 1 in the Supplement). For 22 of 45 subspecialties (49%), women had a lower mean annual salary growth rate than men. The differences in mean annual salary growth rates ranged from 1.2% per year higher for women to 3.1% per year higher for men.

**Table 1.** Differences in Estimated Starting Salary, Year-10 Salary, Mean Annual Salary Growth Rate, and 10-Year NPV Between Female and Male Academic Physicians in the First 10 Years of Posttraining Employment^a^

Subspecialty	Difference, median (IQR), $ [%]^b^			Difference in mean annual salary growth rate, median (IQR), %/y
	Starting salary	Year-10 salary	10-y NPV	
Adult medical	29 854 (22 673 to 39 432) [11]	21 504 (15 900 to 34 621) [8]	227 501 (170 419 to 322 471) [11]	–0.2 (–0.5 to 0.1)
Adult surgical	64 124 (56 849 to 109 613) [17]	99 385 (83 737 to 127 805) [20]	631 739 (533 573 to 1 004 672) [19]	0.5 (–0.1 to 1.0)
Pediatric medical	9632 (620 to 18 008) [4]	18 841 (6957 to 24 215) [7]	108 036 (29 901 to 141 088) [7]	0.3 (–0.2 to 0.9)
All subspecialties	26 800 (12 816 to 40 980) [10]	22 890 (15 808 to 49 781) [9]	214 440 (130 423 to 384 954) [10]	0.0 (–0.4 to 0.6)

Abbreviation: NPV, net present value.

^a^ Positive numbers indicate higher salary, growth rate, or 10-year NPV for men compared with women.

^b^ Salaries are shown in 2019 to 2020 US dollars.

**Figure 2.**
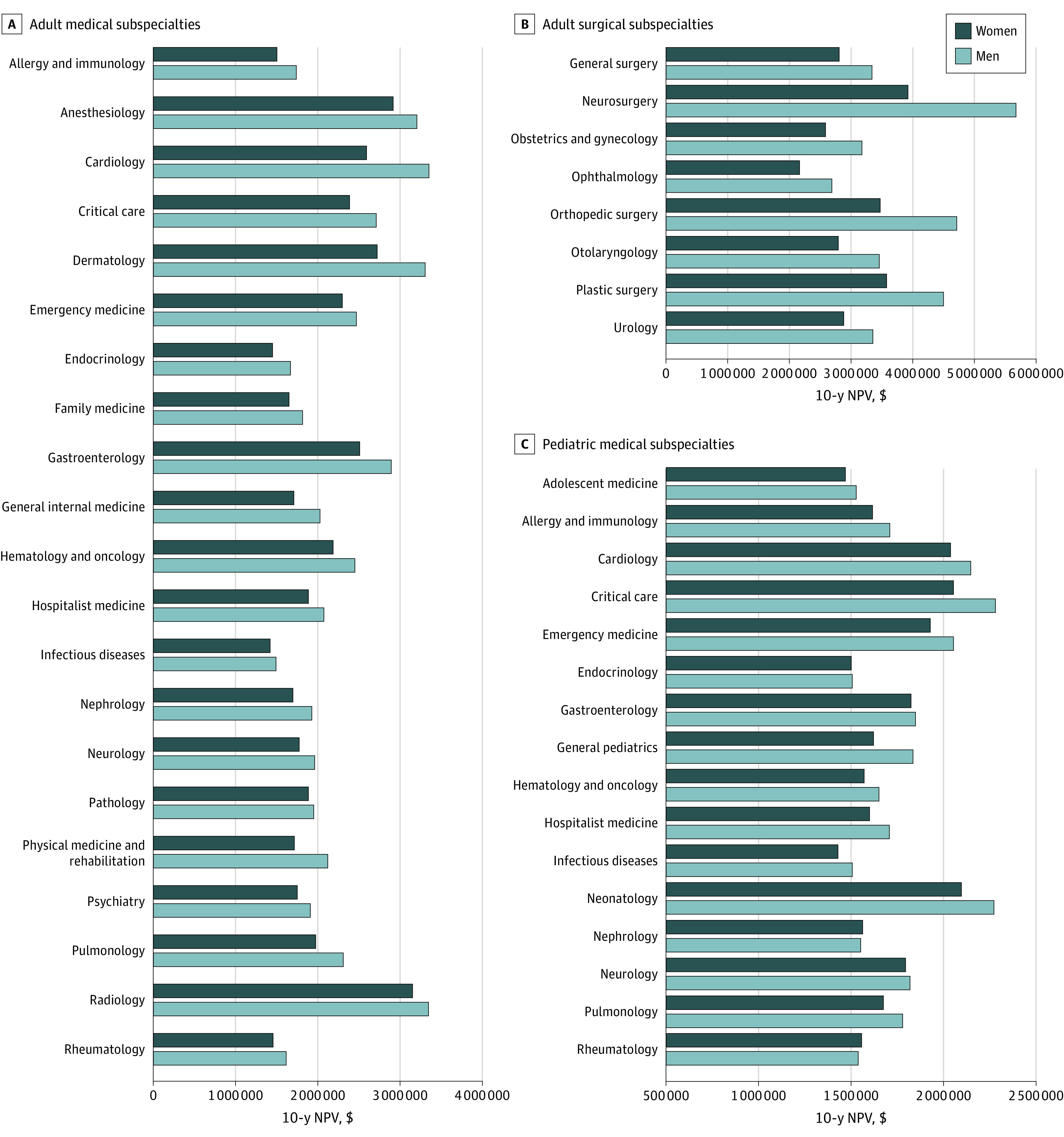
Earning Potential in the First 10 Years of Posttraining Employment by Gender Graphs show data for adult medical (A), adult surgical (B), and pediatric medical (C) subspecialties. NPV indicates net present value.

Female physicians had lower 10-year NPV in 43 of 45 subspecialties (96%) across the adult medical (Figure 2A), adult surgical (Figure 2B), and pediatric medical (Figure 2C) subspecialties (Table 1 and eTable 1 in the Supplement). The 10-year NPV was a median (IQR) of $214 440 ($130 423-$384 954), or 10%, less for women than for men. In only 2 subspecialties, pediatric nephrology and pediatric rheumatology, was the 10-year NPV higher for women.

### Sensitivity Analyses

A 1-year delay in promotion from assistant to associate professor for women compared with men decreased the 10-year NPV of women by a median (IQR) of $26 042 ($35 671-$19 672), or 2% (Table 2 and eTable 2 in the Supplement). Failure to be promoted at all decreased the 10-year NPV of women by a median (IQR) of $218 724 ($176 317-$284 466), or 13%.

**Table 2.** Sensitivity Analyses: Estimated Impact of 1-Year Delay in Promotion and of Failure to Be Promoted From Assistant to Associate Professor for Female Academic Physicians on Estimated Earning Potential in the First 10 Years of Posttraining Employment

Subspecialty	Change in 10-y NPV for women, median (IQR), $ [% change of original 10-y NPV for women]^a^	
	1-y Promotion delay	No promotion
Adult medical	–20 627 (–35 671 to –18 208) [–1]	–201 941 (–225 745 to –170 151) [–10]
Adult surgical	–49 842 (–54 963 to –28 171) [–2]	–261 622 (–314 005 to –171 324) [–12]
Pediatric medical	–26 914 (–32 171 to –20 078) [–2]	–222 866 (–258 592 to –198 988) [–15]
All subspecialties	–26 042 (–35 671 to –19 672) [–2]	–218 724 (–284 466 to –176 317) [–13]

Abbreviation: NPV, net present value.

^a^ Salaries are shown in 2019 to 2020 US dollars.

Equalizing starting salaries for women to those of men could increase women’s 10-year NPV by a median (IQR) of $250 075 ($161 299-$381 799) across the 42 subspecialties in which the starting salaries for women were lower than those for men (Table 3, eTable 2, and eFigure in the Supplement). This represented a median of 106% of the original absolute difference in 10-year NPV between women and men in those subspecialties. The change was greater than 100% because, in this sensitivity analysis, growth rates were held constant; in subspecialties in which the starting salaries for women were lower than for men, but the mean annual salary growth rates were modestly higher for women, the resultant 10-year NPV for women in those fields ended up being slightly higher than those for men, yielding a greater than 100% change.

**Table 3.** Sensitivity Analyses: Estimated Impact of Equalizing Starting Salaries or Equalizing Mean Annual Salary Growth Rates for Female Academic Physicians on Earning Potential in the First 10 Years of Posttraining Employment^a^

Subspecialty	Change in 10-y NPV for women, median (IQR) $ [% change of original difference in 10-y NPV between women and men]^b^	
	Equalizing starting salaries	Equalizing mean annual salary growth rates
Adult medical	280 405 (202 254-349 170) [108]	17 541 (9238-47 073) [6]
Adult surgical	596 690 (524 877-1 018 724) [98]	115 706 (110 182-179 766) [23]
Pediatric medical	116 091 (46 803-166 372) [88]	54 691 (39 226-70 984) [59]
All subspecialties	250 075 (161 299-381 799) [106]	53 661 (24 258-102 892) [21]

Abbreviation: NPV, net present value.

^a^ In 42 of 45 subspecialties, the annual salary in the first year of employment was lower for women than men; these 42 subspecialties were included in the analysis on equalizing starting salaries. In 22 of 45 subspecialties, the mean annual salary growth rate was lower for women than men; these 22 subspecialties were included in the analysis on equalizing salary growth rates.

^b^ Salaries are shown in 2019 to 2020 US dollars.

Adjusting women’s mean annual salary growth rate to match that of men could increase their 10-year NPV by a median (IQR) of $53 661 ($24 258-$102 892) across the 22 subspecialties in which the mean annual salary growth rate was lower for women than for men (Table 3, eTable 2, and eFigure in the Supplement). This represented a median of 21% of the original absolute difference in 10-year NPV between women and men in these subspecialties.

## Discussion

In this cross-sectional study, we used standard financial techniques to estimate the financial returns in the early years of a career in academic medicine in the US for women compared with men. Our results demonstrated wide disparities in early career earning potential between the genders, associated with differences in annual salary that started immediately after the completion of training. Even when annual salary growth rates were similar for women and men in a subspecialty, the differences in starting salary led to a substantial difference in earning potential within the first 10 years of employment. Our results highlight that the issue of unequal compensation between the genders is pervasive and exists within nearly all subspecialties. Although this study focused on gender-based disparities within subspecialties, there are also stark differences in compensation between subspecialties. Prior studies^1,6^ have found that overall compensation for a subspecialty is associated with the proportion of women in its workforce and that, over time, subspecialties that become more female-predominant tend to experience a relative decline in compensation. A recent study of both surgical and nonsurgical subspecialists found that gender differences in income were largest in those practices with the highest proportion of male physicians.^28^

We examined the role of 2 factors associated with disparities in early career earning potential: starting salaries and annual salary growth rates. There are many elements that determine a physician’s annual salary growth rate, such as number of hours worked, clinical productivity, research output, administrative load, and teaching responsibilities.^29^ In fact, some departments simply may institute uniform annual salary percentage increases; it should be noted that these uniform annual salary percentage increases only compound any differences in starting salaries over time. Although annual salary growth rates might vary on the basis of many factors, differences in starting salary seen immediately after the completion of training for new faculty, who are, in theory, equivalent except for their gender, are less easily rationalizable. This is especially true as the data used for this analysis reflected comparative salaries based on full-time employment. In all the adult medical and surgical subspecialties analyzed, women had lower starting salaries and early career earning potential than men. The 3 subspecialties in which women had higher starting salaries and the 2 subspecialties in which women had higher early career earning potential than men were in pediatrics, which is traditionally a lower paying area of medicine.^17^ Our findings are similar to those of Lo Sasso et al,^10^ who examined differences in starting salary for graduating residents in New York State, but we demonstrated that this trend extends nationally and is present within and across subspecialties.

A commonly cited explanation for these differences in starting salary is that women do not negotiate as frequently or as successfully as men.^30,31^ Moreover, women who do try to negotiate often are penalized disproportionately.^32^ Medical school curricula and postgraduate training should better address negotiation skills and financial literacy. However, the onus for ensuring salary equity should not fall on the individual candidate alone; rather, departmental and hospital leadership should take responsibility to ensure uniform starting salaries and prevent gender-based inequalities.

Our analysis found that equalizing starting salaries likely would be more impactful than equalizing annual salary growth rates in terms of raising the early career earning potentials of women and making them closer to those of men. However, in addition to addressing salary parity at the start, periodic compensation evaluations and adjustments are warranted to prevent gender-based divergence in career-long earnings. This is absolutely necessary, both to develop future compensation plans and to address any preexisting gender-based salary inequities for those women currently well into their careers. In addition, we demonstrated how gender-based disparities in promotion timing and occurrence could further exacerbate differences in earning potential. The lack of women in senior ranks and leadership roles in academic medicine has been well documented.^4,13,33,34^ Although our study focused on the early career to minimize the uncertainty in our projections, the financial consequences for women would only be compounded by barriers to promotion and the associated increase in compensation over the duration of their entire careers.

Our results are in keeping with those of other studies that emphasize that addressing gender-based disparities in compensation will require a multipronged approach. The American Medical Association, the American College of Physicians, and other physician groups have recently adopted policies to promote transparency in initial and subsequent compensation, including basing pay on gender-neutral criteria, using training to mitigate bias against women, and implementing routine gender-based pay audits.^13,35^ The shift in the AAMC’s Medical School Faculty Salary Survey report, which started publishing compensation data broken down by gender in 2020, was an important first step in increasing transparency and will allow for regular reevaluations to assess the effectiveness of different interventions. Beyond financial disparities, women face a myriad of potential challenges in academic medicine, including a lack of mentorship, workplace discrimination, and disproportionate duties outside of work related to parenthood or other family caregiving responsibilities.^36^ Addressing many of these issues may have a secondary benefit of improving pay equity. Furthermore, although we focused our analysis on differences within subspecialties, addressing differences in reimbursement for procedural codes, which are primarily used by male-dominated subspecialties, compared with evaluation and management codes, which are used more frequently by female-dominated subspecialties, potentially could mitigate differences in earning potential between subspecialties related to the gender composition of their workforce.^36^

In addition, we have previously demonstrated that there is an association between earning potential and both current and future workforce distribution.^15,16^ Thus, as medicine as a whole and certain subspecialties in particular become increasingly female,^6,9^ disparities in earning potential have the possibility to impact workforce distribution and health care access. Moreover, differences in earning potential may be even more pronounced for women of color and future research will need to investigate the intersectionality of gender and race/ethnicity in determining earning potential as these data become available.

### Limitations

Our study has several limitations. The results are dependent on the assumptions inherent to our models, which include those regarding timing of academic promotion and rates of debt repayment.^15^ We were not able to find data regarding differences in typical debt repayment practices between men and women. In addition, we only used data reflecting practice at academic medical centers. Although gender-based disparities in compensation exist in private practice settings as well,^12^ we believe that academic institutions are the most appropriate initial setting to study because they may have more motivation to address gender-based disparities as part of an evolving focus on equity in their broader mission. Also, given the available data, we were not able to include every subspecialty, examine regional differences, or evaluate the impact of part-time employment. It should be noted that the higher rates of part-time work among female physicians is often cited as a factor associated with their lower earnings^29^; however, the AAMC data we used reflect compensation for full-time employment. Furthermore, using preexisting salary data, such as the AAMC reports on which our models are based, to generate future salary benchmarks can perpetuate existing trends, such as gender-based or subspecialty-based inequalities.

## Conclusions

Our results demonstrate wide differences in starting salary and early career earning potential between female and male academic physicians in the US. The vast majority of these differences in earning potential can be attributed to lower starting salaries for women. Addressing these disparities is necessary to establish equity between health care practitioners. An important next step involves addressing the gender-based disparities in compensation with ongoing measurement and programs that incentivize appropriate action and outcomes. This is vital to ensure equity for physicians and appropriate access to care for patients and their families.

## Notes

Supplement. **eFigure.** Estimated Impact of Equalizing Starting Salaries or Mean Annual Salary Growth Rates Between Women and Men on Earning Potential in the First 10 Years of Posttraining Employment for (A) Adult Medical, (B) Adult Surgical, and (C) Pediatric Medical Subspecialties **eTable 1.** Estimated 10-Year NPV, Starting Salary, Year-10 Salary, and Mean Annual Salary Growth Rate Over the First 10 Years of Posttraining Employment for Female and Male Academic Physicians in the United States **eTable 2.** Results of Sensitivity Analyses on 10-Year NPV for Female Academic Physicians Evaluating the Estimated Impact of Equalizing Starting Salaries, Equalizing Mean Annual Salary Growth Rates, Delaying Promotion From Assistant to Associate Professor by 1 Year, and Failing to be Promoted From Assistant to Associate Professor Within the First 10 Years of a Career
eReference
