# Supplementary material for: Addressing Gender-Based Disparities in Earning Potential in Academic Medicine
Source: JAMA Netw Open. 2022 Feb 18;5(2):e220067. doi: 10.1001/jamanetworkopen.2022.0067 (PMC8857688; doi:10.1001/jamanetworkopen.2022.0067)
Supplement: Supplement. — eFigure. Estimated Impact of Equalizing Starting Salaries or Mean Annual Salary Growth Rates Between Women and Men on Earning Potential in the First 10 Years of Posttraining Employment for (A) Adult Medical, (B) Adult Surgical, and (C) Pediatric Medical Subspecialties eTable 1. Estimated 10-Year NPV, Starting Salary, Year-10 Salary, and Mean Annual Salary Growth Rate Over the First 10 Years of Posttraining Employment for Female and Male Academic Physicians in the United States eTable 2. Results of Sensitivity Analyses on 10-Year NPV for Female Academic Physicians Evaluating the Estimated Impact of Equalizing Starting Salaries, Equalizing Mean Annual Salary Growth Rates, Delaying Promotion From Assistant to Associate Professor by 1 Year, and Failing to be Promoted From Assistant to Associate Professor Within the First 10 Years of a Career eReference [file jamanetwopen-e220067-s001.pdf]

Supplemental Online Content

Catenaccio E, Rochlin JM, Simon HK. Addressing gender-based disparities in earning potential in academic medicine. *JAMA Netw Open*. 2022;5(2):e220067. doi:10.1001/jamanetworkopen.2022.0067

eFigure. Estimated Impact of Equalizing Starting Salaries or Mean Annual Salary Growth Rates Between Women and Men on Earning Potential in the First 10 Years of Posttraining Employment for (A) Adult Medical, (B) Adult Surgical, and (C) Pediatric Medical Subspecialties
eTable 1. Estimated 10-Year NPV, Starting Salary, Year-10 Salary, and Mean Annual Salary Growth Rate Over the First 10 Years of Posttraining Employment for Female and Male Academic Physicians in the United States
eTable 2. Results of Sensitivity Analyses on 10-Year NPV for Female Academic Physicians Evaluating the Estimated Impact of Equalizing Starting Salaries, Equalizing Mean Annual Salary Growth Rates, Delaying Promotion From Assistant to Associate Professor by 1 Year, and Failing to be Promoted From Assistant to Associate Professor Within the First 10 Years of a Career
eReference

This supplemental material has been provided by the authors to give readers additional information about their work.

© 2022 Catenaccio E et al. *JAMA Network Open.*

eFigure. Estimated Impact of Equalizing Starting Salaries or Mean Annual Salary Growth Rates Between Women and Men on Earning Potential in the First 10 Years of Posttraining Employment for (A) Adult Medical, (B) Adult Surgical, and (C) Pediatric Medical Subspecialties. Bars are missing for subspecialties not included in a sensitivity analysis. NPV, net present value; PM&R, physical medicine and rehabilitation.

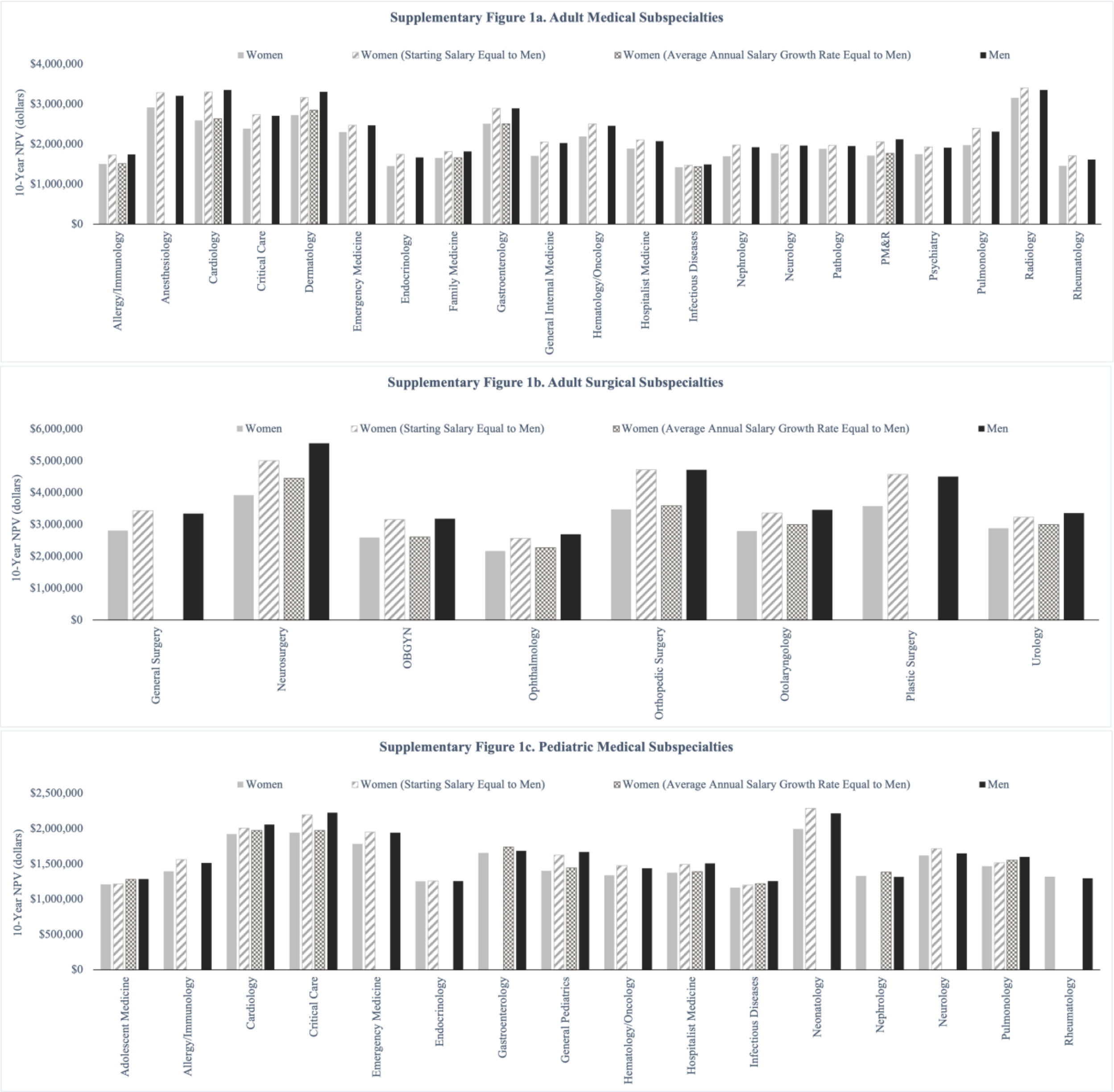

© 2022 Catenaccio E et al. *JAMA Network Open.*

eTable 1. Estimated 10-Year NPV, Starting Salary, Year-10 Salary, and Mean Annual Salary Growth Rate Over the First 10 Years of Posttraining Employment for Female and Male Academic Physicians in the United States. Projections based on cross-sectional mean compensation data by rank for academic physicians from the Association of American Medical Colleges (AAMC) 2019-2020 Faculty Salary Report.[1] NPV, net present value; OBGYN, obstetrics and gynecology; PM&R, physical medicine and rehabilitation.

Subspecialties	10-Year NPV ($)		Starting Salary ($)		Year-10 Salary ($)		Average Annual Salary Growth Rate (%/year)	
**Adult Medical**	Women	Men	Women	Men	Women	Men	Women	Men
Allergy/Immunology	1,500,937	1,740,103	198,988	224,692	210,794	242,315	0.7	0.8
Anesthesiology	2,914,921	3,207,758	350,234	391,213	396,341	419,725	1.3	0.7
Cardiology	2,590,792	3,354,332	321,170	401,773	350,854	454,322	1.0	1.3
Critical Care	2,385,378	2,707,848	295,787	335,219	325,363	360,463	1.0	0.8
Dermatology	2,723,955	3,305,591	320,329	367,793	387,097	490,364	2.0	3.1
Emergency Medicine	2,299,249	2,469,668	285,268	304,599	312,612	333,580	1.0	1.0
Endocrinology	1,450,424	1,664,864	185,968	218,666	216,481	229,311	1.6	0.5
Family Medicine	1,651,709	1,817,055	216,053	234,578	222,511	243,229	0.3	0.4
Gastroenterology	2,510,096	2,895,050	302,289	344,721	356,541	406,321	1.7	1.8
General Internal Medicine	1,708,953	2,024,101	215,640	253,958	240,900	275,521	1.2	0.9
Hematology/Oncology	2,187,277	2,453,732	262,037	296,073	323,475	349,838	2.3	1.8
Hospitalist Medicine	1,886,127	2,075,870	237,613	262,195	266,264	284,912	1.2	0.9
Infectious Diseases	1,421,154	1,492,038	181,168	186,946	215,385	228,002	1.9	2.2
Nephrology	1,696,566	1,924,068	205,521	235,375	261,126	282,078	2.6	1.9
Neurology	1,772,988	1,962,674	223,741	246,414	250,780	272,284	1.2	1.1
Pathology	1,883,633	1,950,623	227,127	236,491	279,901	285,262	2.3	2.0
PM&R	1,715,582	2,120,209	222,885	262,288	234,334	294,751	0.5	1.2
Psychiatry	1,749,175	1,910,778	224,372	245,212	242,392	258,292	0.8	0.6
Pulmonology	1,974,766	2,309,661	245,981	292,961	280,036	306,641	1.4	0.5
Radiology	3,154,553	3,348,944	373,221	400,747	432,941	447,905	1.6	1.2
Rheumatology	1,457,784	1,615,901	181,180	207,980	226,694	231,542	2.4	1.2
**Adult Surgical**								
General Surgery	2,807,393	3,343,293	326,689	393,836	405,596	461,210	2.3	1.7
Neurosurgery	3,923,928	5,674,397	493,986	621,804	485,604	818,584	-0.1	3.0
OBGYN	2,588,086	3,182,928	302,222	363,323	374,413	458,198	2.3	2.5

© 2022 Catenaccio E et al. *JAMA Network Open.*

Ophthalmology	2,164,728	2,691,318	263,785	307,891	306,608	397,779	1.6	2.8
Orthopedic Surgery	3,472,692	4,715,850	400,430	535,428	490,454	653,722	2.2	2.1
Otolaryngology	2,794,644	3,463,281	326,463	387,559	402,256	509,856	2.2	3.0
Plastic Surgery	3,580,993	4,506,170	397,131	500,675	534,769	650,753	3.2	2.8
Urology	2,881,937	3,358,686	350,999	389,933	389,132	472,727	1.1	2.1
**Pediatric Medical**								
Adolescent Medicine	1,211,794	1,286,898	162,540	162,979	184,373	207,264	1.4	2.6
Allergy/Immunology	1,394,655	1,512,894	176,635	194,681	214,575	221,623	2.1	1.4
Cardiology	1,923,437	2,059,627	234,602	243,530	286,874	315,065	2.2	2.8
Critical Care	1,943,831	2,228,087	240,432	267,891	283,316	326,202	1.8	2.1
Emergency Medicine	1,785,868	1,941,649	220,363	238,358	267,369	285,931	2.1	2.0
Endocrinology	1,252,300	1,257,176	164,324	165,004	194,095	194,600	1.8	1.8
Gastroenterology	1,657,221	1,685,400	208,505	202,624	247,005	266,125	1.8	3.0
General Pediatrics	1,403,315	1,668,150	180,507	205,120	203,918	245,741	1.3	1.9
Hematology/Oncology	1,340,470	1,438,301	167,532	181,802	216,721	223,404	2.8	2.3
Hospitalist Medicine	1,375,783	1,508,791	178,887	191,702	208,303	228,631	1.6	1.9
Infectious Diseases	1,163,499	1,257,439	154,240	158,319	183,243	206,078	1.9	2.9
Neonatology	1,996,415	2,216,681	243,156	274,899	295,074	310,882	2.1	1.3
Nephrology	1,328,385	1,316,804	171,130	164,163	206,505	215,080	2.0	3.0
Neurology	1,619,157	1,649,633	196,604	206,940	251,465	243,699	2.7	1.8
Pulmonology	1,469,731	1,600,154	184,597	189,637	228,257	261,166	2.3	3.5
Rheumatology	1,320,906	1,297,672	168,082	166,013	209,208	205,661	2.4	2.3

© 2022 Catenaccio E et al. *JAMA Network Open.*

eTable 2. Results of Sensitivity Analyses on 10-Year NPV for Female Academic Physicians Evaluating the Estimated Impact of Equalizing Starting Salaries, Equalizing Mean Annual Salary Growth Rates, Delaying Promotion From Assistant to Associate Professor by 1 Year, and Failing to be Promoted From Assistant to Associate Professor Within the First 10 Years of a Career. Subspecialties in which the starting salary or the mean annual salary growth rate was higher for women than for men were excluded from those respective analyses (blank rows). NPV, net present value; OBGYN, obstetrics and gynecology; PM&R, physical medicine and rehabilitation.

	10-Year NPV for Women ($)			
**Subspecialties**	One Year Promotion Delay	No Promotion	Equal Starting Salaries	Equal Average Annual Salary Growth Rates
**Adult Medical**				
Allergy/Immunology	1,492,871	1,357,486	1,725,018	1,514,296
Anesthesiology	2,884,060	2,702,101	3,282,066	
Cardiology	2,570,791	2,381,835	3,302,399	2,632,306
Critical Care	2,365,527	2,210,598	2,734,548	
Dermatology	2,678,873	2,349,761	3,160,599	2,850,235
Emergency Medicine	2,280,888	2,129,097	2,470,153	
Endocrinology	1,429,796	1,248,483	1,746,548	
Family Medicine	1,647,336	1,582,456	1,811,500	1,656,825
Gastroenterology	2,473,727	2,284,351	2,896,779	2,508,579
General Internal Medicine	1,691,930	1,534,249	2,050,301	
Hematology/Oncology	2,145,736	1,856,397	2,503,161	
Hospitalist Medicine	1,866,902	1,729,784	2,105,440	
Infectious Diseases	1,398,003	1,202,430	1,473,937	1,438,695
Nephrology	1,658,872	1,388,187	1,976,972	
Neurology	1,754,780	1,596,671	1,975,242	
Pathology	1,847,962	1,593,784	1,970,473	
PM&R	1,707,897	1,633,936	2,058,273	1,768,213
Psychiatry	1,737,024	1,608,145	1,932,344	
Pulmonology	1,951,881	1,786,929	2,396,579	
Radiology	3,114,485	2,870,087	3,403,648	
Rheumatology	1,427,061	1,238,240	1,707,995	
**Adult Surgical**				
General Surgery	2,754,290	2,472,552	3,432,471	

© 2022 Catenaccio E et al. *JAMA Network Open.*

Neurosurgery	3,929,145	1,856,397	5,006,407	4,454,606
OBGYN	2,539,522	1,729,784	3,156,387	2,610,163
Ophthalmology	2,135,796	1,202,430	2,563,950	2,273,850
Orthopedic Surgery	3,412,151	1,388,187	4,722,272	3,586,054
Otolaryngology	2,743,524	1,596,671	3,361,406	2,994,983
Plastic Surgery	3,487,689	3,809,983	4,578,466	
Urology	2,856,051	2,283,565	3,227,543	2,999,986
**Pediatric Medical**				
Adolescent Medicine	1,197,023	1,052,902	1,215,722	1,282,778
Allergy/Immunology	1,369,026	1,184,330	1,561,027	
Cardiology	1,888,026	1,608,793	2,005,940	1,975,155
Critical Care	1,914,852	1,680,067	2,193,770	1,974,631
Emergency Medicine	1,754,117	1,528,999	1,951,689	
Endocrinology	1,232,150	1,058,587	1,258,495	
Gastroenterology	1,631,179	1,415,318		1,741,420
General Pediatrics	1,387,543	1,249,873	1,623,575	1,442,541
Hematology/Oncology	1,307,039	1,058,919	1,475,490	
Hospitalist Medicine	1,355,921	1,194,057	1,491,874	1,391,568
Infectious Diseases	1,143,827	962,753	1,200,743	1,218,734
Neonatology	1,961,395	1,730,176	2,288,989	
Nephrology	1,304,462	1,120,936		1,383,076
Neurology	1,582,111	1,370,624	1,716,578	
Pulmonology	1,440,206	1,234,324	1,516,534	1,551,129
Rheumatology	1,293,120	1,112,736		

eReference:

1. Association of American Medical Colleges. *2019-2020 Faculty Salary Report.* Washington, D.C., 2020.

© 2022 Catenaccio E et al. *JAMA Network Open.*
